# High Temperature Triggers Latent Variation among Individuals: Oviposition Rate and Probability for Outbreaks

**DOI:** 10.1371/journal.pone.0016590

**Published:** 2011-01-27

**Authors:** Christer Björkman, Oskar Kindvall, Solveig Höglund, Anna Lilja, Lars Bärring, Karin Eklund

**Affiliations:** 1 Department of Ecology, Swedish University of Agricultural Sciences, Uppsala, Sweden; 2 Swedish Species Information Centre, Swedish University of Agricultural Sciences, Uppsala, Sweden; 3 Rossby Centre, Swedish Meteorological and Hydrological Institute, Norrköping, Sweden; University of Maribor, Slovenia

## Abstract

**Background:**

It is anticipated that extreme population events, such as extinctions and outbreaks, will become more frequent as a consequence of climate change. To evaluate the increased probability of such events, it is crucial to understand the mechanisms involved. Variation between individuals in their response to climatic factors is an important consideration, especially if microevolution is expected to change the composition of populations.

**Methodology/Principal Findings:**

Here we present data of a willow leaf beetle species, showing high variation among individuals in oviposition rate at a high temperature (20°C). It is particularly noteworthy that not all individuals responded to changes in temperature; individuals laying few eggs at 20°C continued to do so when transferred to 12°C, whereas individuals that laid many eggs at 20°C reduced their oviposition and laid the same number of eggs as the others when transferred to 12°C. When transferred back to 20°C most individuals reverted to their original oviposition rate. Thus, high variation among individuals was only observed at the higher temperature. Using a simple population model and based on regional climate change scenarios we show that the probability of outbreaks increases if there is a realistic increase in the number of warm summers. The probability of outbreaks also increased with increasing heritability of the ability to respond to increased temperature.

**Conclusions/Significance:**

If climate becomes warmer and there is latent variation among individuals in their temperature response, the probability for outbreaks may increase. However, the likelihood for microevolution to play a role may be low. This conclusion is based on the fact that it has been difficult to show that microevolution affect the probability for extinctions. Our results highlight the urge for cautiousness when predicting the future concerning probabilities for extreme population events.

## Introduction

Temperature is important for the distribution and abundance of biological organisms [1]. The increase in temperature during the last decades has resulted in an expansion of the distribution northwards and to higher altitudes for many species [2]–[5]. It is anticipated that climate change may also affect density, possibly leading to a higher incidence of extreme population events, such as extinctions and outbreaks [6]–[13]. However, our ability to judge whether extreme population events will become more frequent in the future depends on how well key processes in population dynamics are understood. For example, it is crucial to know if the target species respond differently to temperature than their host plants/prey and natural enemies [14]–[17]. For example, the reproductive rate of an aphid feeding on pine is both affected directly by temperature and indirectly through changes in host plant quality [18]. Interestingly, this aphid also show higher variability in reproductive rate at high than low temperatures [18], possibly indicating a difference among individuals in their response. Such observations may be an example of phenotypic and/or genotypic variability in temperature response among individuals in a population [13], [19], [20]. If such variation exists, population responses to climate change may be either dampened or enhanced depending on the composition of the population.

The importance for population dynamics of variation among individuals has been emphasised for insects [21], [22] and other organisms [23], [24]. However, there is no empirical evidence to support, for example, the hypothesis that population cycles are driven by oscillating changes in the type of individuals dominating in the population [25], [26]. One reason for the difficulties in demonstrating a connection between variation in individual type and population dynamics could be that individual variation is only expressed under certain environmental conditions [27]. A possible scenario is that if individuals vary in their responsiveness to a change in temperature, the effects of projected temperature increases on the probability of extinctions and outbreaks will be affected by the composition of the population.

Here we focus on insect outbreaks and present evidence that a population of the willow leaf beetle *Phratora vulgatissima* (Coleoptera: Chrysomelidae) consists of individuals that vary with respect to oviposition rate, but this variability is enhanced at higher temperatures. Oviposition rate will be proportional to realised fecundity if the probability for dying varies independently of oviposition rate, as seems to be the case in this system since we have no results indicating that there is a cost connected to high oviposition rate at high temperatures. In population dynamics, variation in realised fecundity is important because increased fecundity is one way by which herbivorous insects escape density dependent regulation and attain outbreak densities [28], [29].

Variation in fecundity among individuals may have genetic or environmental basis, or a combination of both. If we assume that environmental effects are of minor importance, and bear in mind that a life history trait such as egg laying in most cases is to some extent genetically determined [30], we can ask how probability for outbreaks is affected by the heritability of the trait. We analysed this by means of a simple population model in which we allowed microevolution to occur.

As a basis for the modeling we first tested the hypothesis that high temperatures can trigger latent variation in oviposition rate among individual females. This hypothesis was tested by conducting a laboratory experiment with *P. vulgatissima* in which only temperature was varied. The temperatures used in the experiment were related to regional climate change projections representing the SRES A1B greenhouse gas emission scenario [31]. This scenario represents intermediate future emissions and was widely used in the fourth IPCC assessment report [32].

## Materials and Methods

### The leaf beetle

The leaf beetle *Phratora vulgatissima* frequently reach outbreak densities in natural willow stands [33]–[35], as well as in willow plantations used for biomass production, where they are considered pests [36]–[38]. It is univoltine in northern Europe and overwinter as adults in cracks and crevices or under the bark of larger trees [33]. This means that the beetles normally leave the site were they were feeding as larvae. However, the distance travelled is often kept to a minimum and beetles are often seen flying between nearby large trees and willow plantations in the spring and autumn [39]. Both the adults and the larvae feed on the leaves of willows [33].

The beetles used in this experiment came from a willow plantation with high densities of the beetle situated in an agricultural area with several willow plantations northwest of Stockholm (59°40′N, 17°30′E). The population densities had been high for several years in the area [38].

### Oviposition rate

The experiment was performed in 1997 and started by keeping a large number (n>50) of pairs (one female and one male), collected at random from a field population, at a constant temperature (20°C) in the laboratory (80% RH, 22L:2D) for 11–15 days. Each pair of beetles was kept separately on potted *Salix viminalis* plants in cages (diam.: 27 cm, height: 80 cm) and was continuously provided with fresh plants. The beetles were collected before oviposition in the field had started. Two groups (n = 20 pairs per group), with the same mean and variance in egg production, were then selected and randomly designated to either continue at a higher temperature (20°C), or be transferred to a lower temperature (12°C). These conditions prevailed for a further 18 days.

Egg counting in both periods started on day three to avoid including data not representative for the temperature treatment. Thus, eggs were counted during 8–15 effective days in each of these two first periods (I and II). The reason for this variation in number of days was logistical; we had to distribute the counting over several days to get as accurate readings as possible. Since we used average number of eggs laid per day, this variation in number of days should have negligible effects on the results. Some females stopped ovipositing before the end of the experiment, resulting in n = 19 for the 12°C treatment and n = 15 for the 20°C treatments. A number of females continued to oviposit into a third experimental period (III) when temperature again was raised to 20°C (n = 10) or kept at 20°C (n = 13). All females were followed until they died, and total egg production was estimated. Thus, the third period lasted from four to 47 days but only females laying eggs during more than ten days were included in the results.

The temperatures chosen were representative of the lowest (10.2°C) and highest (18.5°C) mean temperature recorded for June (i.e. the period when beetles mainly oviposit), in the area where the study was conducted [40].

### Probability for outbreaks

To analyse how probability for outbreaks may be affected by individual variation in oviposition rate in different temperature scenarios we had to make assumptions. One is that fecundity (in turn assumed to be correlated to oviposition rate) is to some extent genetically determined. This assumption seems reasonable because (1) we have no obvious indications of any strong environmental effects on fecundity and (2) a life history trait such as oviposition rate is commonly genetically determined [30]. To analyse how the probability for outbreaks was affected by the heritability we used a simple population model in which we allowed for microevolution to occur. We assumed the population to be composed of two types of individuals, one that always lay a low number of eggs and one that, in favourable years, lay three time as many eggs. The latter type was assumed to occur at random by 10% in each generation and increase in proportion due to natural selection when the frequency of favourable years is high. Different climate change scenarios were introduced by varying the frequency of favourable years and analyzing the probability for outbreaks. The frequency of favourable years is directly linked to the temperature through a threshold temperature. In this way, the population development scenarios can be linked to projected temperature changes derived from an ensemble of regional climate change scenarios described below.

We used stochastic simulations of an individually based model of population dynamics, which is equivalent to the exponential growth model:
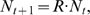



Eqn 1where *R* is the average population growth rate. During the simulation we kept track of each individual and its descendants to successive generations (one generation per time-step). We let a computer determine the number of descendants (*N_i_*), produced by each individual (*i*) randomly from a Poisson distribution with the mean equal to either *F*_normal_ or *F*_good_, which is the average per capita production of descendants surviving to the next generation during normal and good conditions, respectively. *F*_good_ only becomes realised at certain time-steps (favourable years), which occur randomly in time with a specified probability, P(good), and for a certain category of individuals, *i*_good_. Those individuals that on average produce *F*_normal_ descendants per generation independent of current conditions are labelled *i*_normal_. Thus our model deals with two distinct types of individuals that respond differently to random variation in environmental conditions. The population growth rate (Eqn. 1) is equal to the average of all *F*_normal_ and *F*_good_ values realised among the individuals over time.

The individual approach makes it possible to explicitly model various degrees of heritability (*h^2^*) of the two types of individual responses to environmental conditions. The parameter *h^2^* describes the probability that any offspring shares the same trait as its parent. We model this, for each offspring, by generating a random number from a binomial distribution. If an offspring was determined not to share the parents' trait then the type of response was determined randomly with a specified probability, P(type good). Note that this procedure implies no specific assumptions about the mode of heredity or genetic system.

To determine the likelihood of outbreak we carried out 1000 simulations for each combination of parameter settings. Each replicate simulation was initiated with 100 individuals. The fraction of outbreaks was then scored as the proportion of the simulated time-series where the population size reached 1000 individuals within 50 generations, which can be regarded as an appropriate time-horizon when discussing climate change impacts on insect populations [18]. Other parameter values are *F*_normal_ = 1, *F*_good_ = 3 and P(type good) = 0.1.

### Linking the simulations to regional climate change scenarios

During the period 15 May to 13 June, when the willow leaf beetle normally lay eggs, oviposition is favoured by high temperatures. From an empirical analysis of the beetle activity and mean temperature in our research area south of Uppsala and west of Stockholm we found some support for a critical average temperature to be approximately, *T_obs_* = 16°C. This particular critical temperature was chosen for two reasons: (1) Preliminary results from an ongoing laboratory study show that there is no difference in variance (F = 0.57, p = 0.24) between individuals experiencing 16°C and individuals held at 20°C with respect to oviposition rate despite a significant difference between means (t = 5.45, p<0.001, df = 33; mean = 15.8 and 25.7 eggs per day, respectively). (2) During the period 1961–2005, this temperature is rarely reached (only twice) in the study area. While this temperature is favourable for the oviposition rate, it is thus perceived as high by the current beetle population.

The regional climate model RCA3.0 [41], [42] has in the setup used here a resolution of approximately 50 km ×50 km and thus aggregates the regional climate at a much coarser scale than the local climatic variability within the study region. To bridge this gap in spatial scales we used the following four step downscaling and calibration procedure, which based on the method developed by Déqué [43]: i) We analysed temperature data for the study area from a high-resolution gridded database PTHBV (i.e. a gridded dataset of daily mean temperature and total precipitation at 4 km resolution that covers Sweden) to determine the percentile *P_obs_* corresponding to *T_obs_* during the reference period 1961–2005. ii) The *P_obs_* value was then used to find the corresponding temperature *T_RCM_* in the regional climate model experiments for the same period (15 May to 13 June, 1961–2005). To assess the uncertainties and systematic errors in the regional model we first analysed a control experiment produced by using what is called ‘perfect boundary conditions’. The forcing data for this experiment was taken from the European Centre for Medium range Weather Forecasts (ECMWF) reanalysis product ERA40 [44]. iii) The same procedure as in ii) was then applied to the same reference period of ten regional climate change scenarios. These scenarios were run with six different global climate models (GCMs) and come from the Rossby Centre ensemble of regional climate change scenarios [45]. The selected simulations are all forced by the emission scenario A1B from SRES [31]. And finally, iv) we express the temperature dependent probability, *P_ex_*, for future outbreaks as the probability of exceeding *T_RCM_* in the future, i.e. *P_ex_* = 100-*P_scen_* where *P_scen_* is the percentile of *T_RCM_* in the future scenario periods 2011–2040, 2041–2070, 2071–2100. Steps iii) and iv) were repeated for all ten regional climate scenarios and the results were averaged to get an ensemble mean. This ensemble mean gives together with the ensemble standard deviation and range (maximum and minimum) a general picture of the projected possible future of willow leaf beetle populations in an A1B world, assuming micro-evolution to occur. The selected scenarios covers variations induced by having different driving GCMs (BCM, CCSM3, CNRM, ECHAM5, HadCM3, IPSL), different climate sensitivity for one model (HadCM3-Q0 (reference), -Q16 (high), -Q3 (low)), and different initial conditions for another model (ECHAM5-r1, ECHAM5-r2, ECHAM5-r3). In this study we combine all these scenarios to focus on the overall picture of future scenarios of willow leaf beetle population development related to the A1B emission scenario. For a more thorough discussion of various sources of uncertainty see for example Kjellström et al. [45] and Déqué [46] who analyse in detail differences between these regional scenarios.

## Results

### Oviposition rate

**Figure 1:**
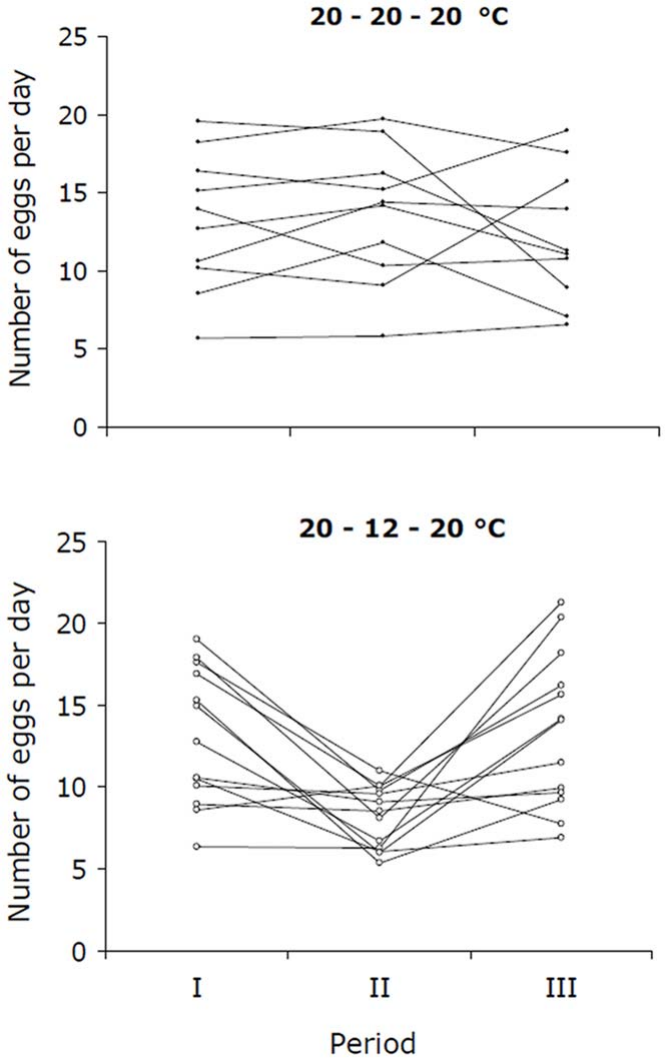
The oviposition rate of individual beetles varies more at high temperature than at low temperature. Average number of eggs laid per day by individual *Phratora vulgatissima* females kept either at constant temperature (20–20–20°C) during all three experimental periods (upper graph) or transferred from high to low and then back to high temperature (20–12–20°C).

On average, female *P. vulgatissima* kept at constant temperature during the whole experimental period (20-20-20°C) continued to lay approximately the same number of eggs in all three periods (Fig. 1 upper; mean(±S.E.)  = 13.1(±4.4) – 13.6(±4.4) – 12.2(±4.3)). The females experiencing low temperature in period II (20–12–20°C) reduced the number of eggs laid per day in this period compared to the number of eggs laid in period I and III (Fig. 1 lower; mean(±SE)  = 13.0(±4.2) – 8.2(±1.9) – 13.4(±4.7)). However, individual females varied considerably in their response. Among females experiencing low temperatures in period II, the variation (i.e. variance) was reduced considerably from period I to II (F_12,12_ = 4.74, p<0.01) and increased from period II to III (F_12,12_ = 6.14, p<0.01). No significant difference in variance was found between any periods for females kept constantly at 20°C or between period I and III for females experiencing 12°C in period II.

It should be noted that not all females experiencing 12°C in period II reduced the number of eggs laid per day, rather it was mainly females laying many eggs per day that did so, whereas most females with low oviposition rate in period I continued to lay few eggs per day in period II (Fig. 1). This pattern is reinforced when using data from all females, including the ones not laying any eggs in period III: There was a significant, positive relationship (r^2^ = 0.781, p<0.001, n = 15), with a slope close to one (y = 0.93x+0.46), between mean number of eggs laid in period I and period II for females kept constantly at 20°C. That is, females that laid many eggs in period I continued to do so in period II, and females that laid few eggs in period I continued to do so in period II. No such relationship (r^2^ = 0.186, p>0.05, n = 19) was found in the group transferred to 12°C, i.e. much of the variation among females in this group disappeared when they experienced the lower temperature, and all females laid a similar number of eggs. This means that females with a high potential oviposition rate were more affected by a reduction in temperature than females with a low potential oviposition rate.

The total number of eggs laid by individual females was not affected by temperature treatment (t = 0.34, p = 0.74): mean total number of eggs (±S.E.) was 591 (±83) for females in the 20–20–20°C treatment and 557 (±56) for females in the 20–12–20°C treatment.

### Probability for outbreaks

**Figure 2:**
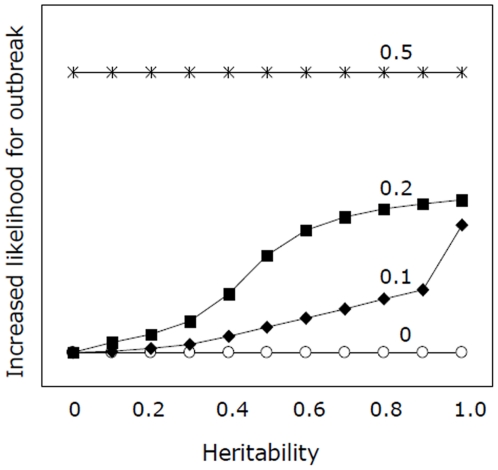
Probability for outbreaks increase with level of heritability and frequency of warm summers. Results from a model describing the relationship between heritability of the trait to be able to lay more eggs at high temperatures and the probability for outbreaks among leaf beetles feeding on willows. How this relationship is affected by the frequency of warm summers, corresponding roughly to different global warming scenarios, is presented; 0 =  no change in climate, 0.1 = 10% of the summers are so warm that individuals with the ability to substantially increase the number of eggs they lay per day can express their maximum potential, 0.2 = 20% of the summers are that warm, and 0.5 = 50% of the summers are that warm. It is assumed in the model that the ability to lay many eggs is determined by one allele in a single locus, and that the allele frequency in the population at start is low.

The model shows that the likelihood for outbreak increases with heritability and the frequency of favourable years. For example, at intermediate levels of heritability, the likelihood for outbreak increase ten times if there are ten (P(good) = 0.20) rather than five (0.10) favourable years within a 50 year period (Fig. 2). So far we have no measurements of the heritability of leaf beetle oviposition.

### Future temperature change

The threshold temperature *T_obs_* corresponds to the percentile *P_obs_* = 98% in the PTHBV data set. In the ERA40 control experiment *P_obs_* percentile corresponds to the threshold temperature *T_RCM_* = 14.7°C. The negative bias, *T_RCM_-T_obs_* = −1.3°C, is a combination of systematic bias in RCA3.0 and the mismatch between the regional climate represented by RCA3.0 and the local climate of the open landscape represented by the PTHBV data set. This mismatch is natural since the regional average represented by RCA3.0 also includes the 2 m temperature inside forests and over lakes which at this time of year is relatively cold. Turning to the reference period of the climate change scenarios we note that the ensemble mean *T_RCM_* = 14.2°C has an additional negative bias of −0.5°C compared to the ERA40 *T_RCM_* = 14.7°C. This additional bias is related to the GCMs, where HadCM3 is consistently somewhat warmer (irrespective of climate sensitivity setting) but the others are cold, in particular IPSL which has *T_RCM_* = 12.5°C. Using these individual threshold temperatures that take the specific biases of each regional scenario into account, the ensemble average changes in probability *P_ex_* undergo substantial changes (Table 1) along with the projected temperature increase. We note that the CCSM3 driven scenario exhibits a rather different time evolution compared to the other scenarios. Contrary to all other scenarios, after having followed a similar general trend as the other scenarios until the last period, 2071–2100, when it to present days level of *P_ex_*. There is no objective argument for excluding this scenario from the analyses, for all but the last period the effect of including or removing the CSM3 driven scenario was however neglible. In Table 1 we therefore present ensemble statistics both with and without this scenario for the last period. There is no difference in median between the two alternatives, and the difference in mean is small. The present day (1961–2005) probability of 2% changes to 9% in the coming decades (2011–2040), increasing to 244% in the middle of the century, and approaches a probability of almost 43% (45% omitting CCSM3) towards the end of the century (2071–2100). As is expected the spread among the scenarios increases with time both in standard deviation and in range. In the coming decades it ranges from 3% to 13%, in the middle of the century it has increased to range from 13% to 33%. Towards the end of the century the ensemble is projected range from 34% to 67% or, including the outlying CCSM3 driven scenario, from 12% to 67%.

**Table 1:** Summary of the regional climate change scenarios.

Driving GCM	Exp.	*T_RCM_* (°C)	*P_ex_* (%)			
		1961–2005	2011–2040	2041–2070	2071–2100	
ERA40, control (44)		14.7				
BCM [47], [48]		15.2	3	13	34	
CCSM3 [49]		13.1	13	27	12	
CNRM [50]		15.0	6	18	32	
ECHAM5 [51], [52]	r1	14.1	4	15	58	
	r2	13.9	11	27	67	
	r3	13.9	10	33	53	
HadCM3 [53]	Q0 (ref)	15.4	13	28	41	
	Q16 (high)	15.5	11	21	45	
	Q3 (low)	13.5	6	29	36	
IPSL [54]		12.5	12	26	54	
Ensemble mean		14.2	9	24	43	(47)
Ensemble median		14.0	10	26	43	(45)
Ensemble standard deviation		1.0	4	7	16	(12)
Ensemble maximum		15.5	13	33	67	(67)
Ensemble minimum		12.5	3	13	12	(32)
Ensemble span		3.0	10	20	55	(35)

*T_RCM_* is the threshold temperature in the regional climate scenarios that corresponds to *T_obs_* = 16°C during the control period 1961–2005. This is the 98th percentile, which also means that the threshold is exceeded in 2% of the cases during the reference period. Columns *P_ex_* is the probability of exceeding this threshold in the future scenario periods. The ERA40 control simulation only covers the control period and is not included in the ensemble summary statistics. Because the CCSM3 driven scenario exhibits a rather different time evolution towards the end of the century compared to the other models, we present ensemble statistics including this scenario included, and in within parentheses also ensemble statistics excluding the CCSM3 driven scenario. Column “Exp.” refer to different experiments with the same GCM; this is explained in section Data and Methods.

## Discussion

By manipulating the temperature experienced by *Phratora vulgatissima* females, we found evidence for the hypothesis that high temperature can trigger latent variation in egg-laying capacity among insect individuals. The systematic difference among individuals in their response to temperature, i.e. ‘low oviposition rate’ individuals being insensitive to temperature change and ‘high oviposition rate’ individuals being responsive has, to our knowledge, not been reported previously. The probability for extreme population events such as outbreaks will depend on the conditions, such as (1) the proportion of individuals in the population with the ability to respond positively to temperature, (2) whether there are costs associated with an increased ovipotion rate, (3) the heritability of the ability to respond positively to increased temperature, (4) how other trophic levels respond to the same change and (5) the frequency of periods with high temperature.

It seems reasonable to conclude that the probability for outbreaks is influenced by the proportion of individuals in the population with the ability to respond to increased temperature by increased oviposition. This conclusion is based on an assumption that requires qualification, i.e. all adult females face the same probability of dying irrespective of whether they have the ability to respond positively to increased temperatures or not. In other words, we assumed that oviposition rate was directly proportional to realised fecundity under natural conditions. If this assumption is correct, females with the ability to respond positively to temperature increase would lay more eggs in total than those lacking this ability. This basic assumption needs to be substantiated, especially as it seems unlikely that no costs are associated with this trait cf. Roff [30] but see Cam et al. [55]. However, so far we have had no indication of such costs. For example, the total number of days that *P. vulgatissima* females laid eggs, i.e. longevity, was not related to number of eggs laid per day (r = −0.098, p = 0.76, n = 12 for females held at a constant temperature and r = −0.336, p = 0.29, n = 12 for females moved to lower temperature). The lack of relationship between longevity and eggs laid per day supports the model assumption that the population growth rate is proportional to the number of eggs laid per day.

Variability among individuals, with respect to other characters possibly correlated to outbreak probability, has been observed, e.g. supercooling point [56], pupal development rate [57] and fecundity [58]. In several of these cases the variation has been shown to be temperature sensitive and/or under genetic control. It is not known to what extent the leaf beetles' ability to respond to changes in temperature by varying the number of eggs laid per day is genetically determined. If some genetic control is assumed, it becomes evident that the probability of outbreak will be affected by (a) the initial proportion of individuals with the ‘trait’ and (b) the heritability of the ‘trait’. Thus, if the frequency of warm years increases then the probability of outbreaks could increase through microevolutionary changes in the leaf beetle population. However, heritability needs to be above 0.3 at least to give a substantial increase in the likelihood for outbreaks. Values of heritability of around 0.4 have been reported for similar traits in insects [30] but we have no such estimates for *P. vulgatissima*.

It can be argued that the model used here is simplistic, e.g. assuming only two types of individuals and not taking into account that variation is continuous. However, our focus is on how probability for outbreaks may change and how this probability varies with heritability, an issue that is captured by the model. A more serious problem with the model could be the assumption that no costs are associated with the ability to respond positively to increased temperatures. Even though it seems reasonable that such costs exist [30], which could explain why the trait has not been fixed in the population, our data do not indicate that they do. For example, longevity of females varied irrespectively of eggs laid per day. An alternative explanation for why the trait has not been fixed in the leaf beetle populations is that they have not experienced warm summers at a sufficiently high frequency.

As there are no other studies on how microevolutionary change may affect the probability for outbreaks in pest species we turn to the other end of the same basic ecological principles, i.e. probability for extinctions. At present there seem to be consensus that microevolutionary change in response to rapid climate change is not likely to function as a mechanism for preserving threatened species [13], [59]. Gienapp et al. [59] conclude that “many responses perceived as adaptations to changing environmental conditions could be environmentally induced plastic responses rather than microevolutionary adaptations”.

Another aspect that needs to be considered when evaluating probabilities for extreme population events is how other trophic levels (e.g. host plants and natural enemies) respond to the same environmental change [13], [16], [17]. Thus, our ability to predict the probability for outbreaks (and extinctions) in relation to climate change would improve if we incorporated trophic interactions to a larger extent in future studies.

The frequency of periods with warm enough periods will also affect the probability for extreme population events. A robust result from the ensemble summary statistics is that the probability of periods exceeding the critical average temperature for triggering the oviposition rate of the willow leaf beetle is increasing in the future. This is based on a ten member ensemble representing the SRES A1B intermediate greenhouse gas emissions. Presently, overall mean temperature above the threshold 16°C during the whole egg-laying period is infrequent. In the coming decades it is projected to occur once in ten years, and towards the middle of this century once in four years. And towards the end of this century the threshold exceedance is projected to occur almost once every second year. Associated with these increasing ensemble averages the spread among the ensemble members also increases with time. These results are based on the assumption that the observed threshold is 16°C. Small changes to the threshold temperature will change the numeric results to some degree, but if the threshold temperature changes enough to be totally outside the observed range, i.e. the precentile for its occurence ‘saturates’ (i.e. reaches 0%), then a warming during the coming decads will cause no change at all before the threshold is reached.

It is of course difficult to argue that a specific temperature (here 16°C) is the critical threshold at which a population starts to behave qualatively differently than it would do at lower temperatures. Still, we believe that the approach used here captures the essence of what could be expected of populations consisting of individuals that vary in their temperature response in a climate change perspective.

Our ten member ensemble samples several aspects of the uncertainty inherent in climate modelling, i.e. variations induced by employing different forcing GCMs, variation in climate sensitivitiy and due to different initial conditions. Other aspects like diffferent SRES emission scenarios are not included in this study. As we do not know about the future it is impossible to determine which of the regional climate scenarios that gives the most ‘accurate’ representation of the future conditions. A good starting point, to get a general picture of how the future probability of insect outbreaks is developing, is to study the ensemble mean and the associated spread. Based on such information about climate we could improve our ability to predict and circumvent extreme population events, such as outbreaks, in future forests and agriculture by increasing our basic understanding of key population processes involved.

A warmer climate will lead to more migration and dispersal [4], [7] that, in turn, will affect the composition of populations. In insect species more well-studied than *P. vulgatissima* it has been documented that weather conditions affect the way sub-populations with different reproductive strategies intermix [60]. Whether such a phenomenon exist in *P. vulgatissima* remains to be documented but could partly explain observed pattens.

Albeit obvious simplifications in the population model and insecurities in the climatic scenarios we are confident in our conclusion that the risk for outbreaks will depend on the proportion of individuals with the ability to respond positively to increasing temperatures. The role of individual variation in life history traits for the occurrence of extreme population events needs to be investigated further if we aim at making better predictions of how climate change might affect the probability for outbreaks and extinctions.
